# Factors Influencing Delivery of Cancer Survivorship Care Plans: A National Patterns of Care Study

**DOI:** 10.3389/fonc.2019.01577

**Published:** 2020-01-31

**Authors:** Joseph L. Benci, Carolyn C. Vachani, Margaret K. Hampshire, Christina Bach, Karen Arnold-Korzeniowski, James M. Metz, Christine E. Hill-Kayser

**Affiliations:** Department of Radiation Oncology, Perelman Center for Advanced Medicine, University of Pennsylvania, Philadelphia, PA, United States

**Keywords:** survivorship, survivorship care plans, cancer, health disparities, cancer survivor

## Abstract

Nearly half of all Americans will develop cancer at least once in their lifetime. Through improved screening and treatments, the number of cancer survivors is reaching all-time highs. However, survivorship care plans (SCPs) are inconsistently used, denying many survivors access to critical information. This study used 46,408 SCPs generated from 2007 to 2016 and applied machine learning to identify predictors of SCP creation, including cancer type, type of physician, and healthcare center where they received care, as well as regional variations in care plan creation. Identifying these disparities in SCP use is a critical first step in efforts toward expanding access to survivorship care planning. Using a convenience sample of survivors, it is possible to model the factors that predict generation of SCPs either by the survivor or by a healthcare provider. This study identifies several important disparities both survivor intrinsic such as cancer type, as well as treatment associated and geographic differences in SCP generation. Identifying these disparities at the national level across cancer types will allow for more targeted recommendations to improve SCP creation and dissemination in underserved groups.

## Introduction

Cancer survivors represent a heterogeneous population spanning all ages, genders, ethnicities, and income levels. Through improved screening and detection as well as therapeutic advancements, they are also one of the fastest growing patient populations. The United States alone is expected to have 18 million living cancer survivors by 2022 (1), a group larger than the entire population of the Netherlands. Such a large and diverse patient group unsurprisingly has a diverse array of needs: stemming from physical side effects from of their cancer treatments, psychological disorders such as depression or anxiety, or economic issues from lost productivity or medical bills related to their individual cancer treatment or as a result of normal aging.

To help address these issues, in 2005 the Institute of Medicine recommended that all patients completing cancer treatment receive a survivorship care plan (SCP) which contains information about the treatments the patient received, possible side effects stemming from those treatments, recommendations for continued cancer screening, information about follow-up care, and general wellness tips (2). Patients who received a SCP at the end of treatment were more likely to have increased contact with their primary care physician (3, 4), and reported improved patient-provider communication (5). This improved communication and contact with healthcare providers is important because it increases the likelihood a survivor will be screened and catch a recurrence or other side effect early. In addition to more frequent and better interactions with their healthcare provider, survivors who received a SCP were also less likely to report experiencing depressive symptoms both in the short-term and long-term; this benefit was maintained even for survivors who were more than 5 years post-diagnosis (6).

Despite the numerous benefits of receiving a SCP and recommendations by the Institute of Medicine and American College of Surgeons Commission on Cancer that all cancer survivors receive one, most healthcare centers do not consistently provide them to patients (7). A study of all 51 National Cancer Institute (NCI) designated cancer centers found that plans were predominantly used for breast and colorectal cancer survivors (8). Another study of 81 cancer centers found SCP use was positively associated with academic medical centers and negatively associated with freestanding medical centers (9). However, little is known about the patient-level factors that influence SCP use. One study of gynecological cancer survivors found that older patients and those with ovarian cancer were less likely to receive a SCP (10), while another study on skin cancer found that older survivors were more likely to receive care plans (11). While each of these studies was focused on a narrowly defined survivor population, it is unclear how patient and treatment related characteristics affect SCP creation across the overall cancer survivor population.

This study utilized a nationwide convenience sample of survivors, family members, and health care providers who utilized a free, online tool to generate SCPs across all cancer types. We utilized this information in combination with supervised machine learning to identify which factors influence whether a survivor would create or have a SCP created for them. Exploring current patterns of care plan use is an important first step to identifying underserved survivor populations in order to improve survivor access to SCPs.

## Materials and Methods

### Survivorship Care Plans

OncoLink, an Internet-based survivorship resource at the University of Pennsylvania, developed and maintains a free, publicly available online SCP generator (www.oncolink.org) (12). The interactive online survivorship resource enables health care providers, patients, and/or their family members to input demographic data, information on cancer type and status, treatments, and information about the type of physician managing care and the cancer treatment setting. An in-depth explanation of the questions used to generate the SCP and information contained in the OncoLink resource has been previously reported (12). Based on the cancer and treatment information, the user then receives personalized, evidence-based health care recommendations for future care, potential long term side effects, and other general wellness suggestions.

### Data Collection and Inclusion Criteria

All patients, family members and providers who used Oncolink from its inception in 2007 to December 2016 were included in this analysis. Responses from both the provider version of the resource, where providers enter the information on behalf of patients to provide them with the resulting care plan, and the version patients and family members can access directly were included. Institutional Review Board approval was obtained before data collection and subsequent analysis.

Only users who completed all questions and generated a SCP were used in this analysis. Patients and family members who indicated they were unsure if they had previously received a care plan were excluded. Patients or family members who accessed the healthcare provider version of the resource were excluded from the analysis. Only care plans for survivors from the United States were included in the final sample. Both survivor/family member and physician completed SCPs were included in this analysis to represent overall access to survivorship care planning materials, rather than those who just received the information from their healthcare provider.

### Random Forest

Random Forest, a supervised machine learning approach, was used to model the interaction between demographic and treatment associated factors on whether or not a patient received a SCP (13). Random forest was selected based on its ability to handle large data sets of both discrete and continuous variables, its ability to correct for potential covariance between variables within the data set, and the ability to learn the relative importance of each variable on the outcome classification (whether or not a survivor created/had a SCP created for them or not).

A full list of the variables used in this analysis can be found in Supplemental Table 1. The outcome variable for the study was whether a patient had received a SCP, which was a binary “yes” or “no.” On the patient version of the questions used to generate the SCP, participants were asked whether they had previously been provided survivorship care and were categorized as “yes” or “no” based on their response. For the provider version of the questions used to generate the SCP, where providers enter the information on behalf of patients to provide them with the resulting care plan, all respondents were considered “yes” for the purpose of this analysis.

To prevent an over-representation of care plans for certain cancer types from biasing the analysis, the input sample for each tree in the random forest was weighted using the National Cancer Institute's cancer prevalence of the top fifteen most commonly diagnosed cancer types in the United States (14).

The dataset was split into a randomly selected two-thirds of the total responses for use in building the Random Forest model and was then validated on the remaining one-third to provide an estimate of the accuracy of the model. Analysis was performed using the *randomForest* R package, with 1,000 trees per run, node size of 1, mtry set to 23, and 4,000 randomly selected care plans per tree.

### Univariate Statistical Analyses

To validate results from the Random Forest, univariate analysis by chi-squared test was performed. Prior to the univariate analysis the complete dataset was randomly subset five times with replacement, weighted on the cancer incidence as discussed above, to create a sample dataset for downstream analysis.

For geographical analyses, the state the respondent indicated they were from was binned into Northeast, South, Midwest, or West based on the geographic designations used by the United States Census Bureau. Data were visualized using the *maps* and *ggplot2* packages in R.

All data analyses were done using the R language and environment for statistical computing and graphics (https://www.r-project.org). All *p*-values are from the Chi square test.

## Results

### Demographics

Survivorship care plans were created for a total of 46,408 survivors (Table 1). Three-quarters of survivors were female and nearly 80% were Caucasian. Survivors were more likely to be from urban or suburban areas, but were nearly evenly split between the different regions of the United States. Over 75% of survivors had their care managed by either an oncologist or the combination of an oncologist and another physician. Care plans for breast cancer survivors made up nearly half of the total number of care plans while other common cancer types including lung and colorectal made up only 5 and 7% of the total, respectively (Supplemental Table 2). Due to the overrepresentation of breast cancer survivors in the OncoLink dataset, all downstream univariate analyses were done with a random subset of the full OncoLink dataset, with each cancer type weighted proportionally to its incidence using the NCI's SEER Cancer Statistics Review of the top fifteen most common cancers in the United States (14). Full demographic data for survivors included in this study can be found in Table 1.

**Table 1:** Characteristics of OncoLink users split by receipt of survivorship care plans (SCPs).

**Received survivorship care plan**	Yes	No
*n*	25,890	20,518
**Sex (%)**		
Male	6,021 (23.3)	5,206 (25.4)
Female	19,869 (76.7)	15,312 (74.6)
**Race (%)**		
Caucasian	20,149 (77.8)	17,374 (84.7)
African American	1,960 (7.6)	1,240 (6.0)
Asian	701 (2.7)	589 (2.9)
Hispanic/Latino	1,347 (5.2)	894 (4.4)
Mixed race	123 (0.5)	193 (0.9)
Other	1,607 (6.2)	228 (1.1)
Diagnosis age (years) [mean (sd)]	40.01 (14.54)	36.30 (14.07)
Time since diagnosis (years) [mean (sd)]	1.96 (4.22)	2.73 (5.45)
**Education (%)**		
< College	6,394 (24.7)	7,973 (38.9)
College degree	3,763 (14.5)	5,923 (28.9)
Graduate degree	1,711 (6.6)	4,181 (20.3)
Not available	14,022 (54.2)	2,441 (11.9)
**Developed environment (%)**		
Rural	4,832 (18.7)	3,326 (16.2)
Suburban	10,214 (39.5)	7,146 (34.8)
Urban	8,899 (34.4)	5,771 (28.1)
Not available	1,945 (7.5)	4,275 (20.8)
**Region of United States (%)**		
Northeast	5,591 (21.6)	5,293 (25.8)
South	7,474 (28.9)	6,084 (29.7)
Midwest	6,160 (23.8)	4,470 (21.8)
West	6,665 (25.7)	4,671 (22.8)
**Cancer situation (%)**		
Metastatic	779 (3.0)	1,082 (5.3)
Recurrence/2nd Cancer	1,516 (5.9)	1,078 (5.3)
Neither	17,282 (66.8)	10,816 (52.7)
Not available	6,313 (24.4)	7,542 (36.8)
**Treatment environment (%)**		
University based cancer center	6,698 (25.9)	4,454 (21.7)
Non-University based hospital cancer center	12,439 (48.1)	7,663 (37.4)
Private doctor's office	2,828 (10.9)	2,603 (12.7)
Combination of these	1,980 (7.7)	1,523 (7.4)
Not available	1,945 (7.5)	4,275 (20.8)
**Managing healthcare (%)**		
Oncologist	8,714 (33.7)	9,220 (44.9)
Primary care physician	2,159 (8.3)	2,736 (13.3)
Combination of these	14,399 (55.6)	7,588 (37.0)
Other	618 (2.4)	974 (4.7)
Distance from treatment center (miles) (%)		
≤20	17,852 (69.0)	11,253 (54.8)
>20	6,093 (23.5)	4,990 (24.3)
Not available	1,945 (7.5)	4,275 (20.8)
**Treatment summary offered (%)**		
Yes	4,608 (17.8)	2,318 (11.3)
No	14,603 (56.4)	12,901 (62.9)
Unsure	6,679 (25.8)	5,299 (25.8)
**Had surgery (%)**		
Yes	21,295 (82.3)	16,666 (81.2)
No	4,595 (17.7)	3,852 (18.8)
**Had IV chemotherapy (%)**		
Yes	17,309 (66.9)	14,777 (72.0)
No	8,581 (33.1)	5,741 (28.0)
**Had intrathecal chemotherapy (%)**		
Yes	289 (1.1)	395 (1.9)
No	25,601 (98.9)	20,123 (98.1)
**Had radiation therapy (%)**		
Yes	16,720 (64.6)	11,572 (56.4)
No	9,170 (35.4)	8,946 (43.6)
**Had stem cell or bone marrow transplant (%)**		
Yes	320 (1.2)	306 (1.5)
No	23,625 (91.3)	15,937 (77.7)
Unsure	1,945 (7.5)	4,275 (20.8)
Number of chemotherapies [mean (sd)]	1.77 (1.84)	1.92 (1.90)
Number of surgeries [mean (sd)]	1.61 (1.19)	1.60 (1.29)
Number of radiation sites [mean (sd)]	1.17 (0.99)	0.85 (0.99)
Number of treatment modalities [mean (sd)]	2.14 (0.76)	2.10 (0.80)
Number of side effects experienced [mean (sd)]	0.42 (1.71)	2.74 (3.55)
**Cancer type (%)**		
Bladder	169 (0.7)	194 (0.9)
Breast	14,106 (54.5)	8,824 (43.0)
Colon	1,698 (6.6)	1,572 (7.7)
Head and neck	1,153 (4.5)	894 (4.4)
Leukemia	413 (1.6)	559 (2.7)
Liver	43 (0.2)	52 (0.3)
Lung	1,450 (5.6)	953 (4.6)
Lymphoma	1,027 (4.0)	1,145 (5.6)
Melanoma	217 (0.8)	324 (1.6)
Other	1,857 (7.2)	2,475 (12.1)
Ovarian	1,585 (6.1)	1,732 (8.4)
Pancreatic	129 (0.5)	174 (0.8)
Prostate	1,493 (5.8)	981 (4.8)
Renal	158 (0.6)	202 (1.0)
Stomach	70 (0.3)	54 (0.3)
Thyroid	322 (1.2)	383 (1.9)

### Using Machine Learning to Identify Factors That Affect Whether a Patient Receives a Survivorship Care Plan

Several differences were identified between survivors who did or did not receive a SCP (Table 1). Breast cancer survivors were much more likely to receive a SCP, while melanoma survivors were less likely to report receiving a plan. Of survivors whose education level was known, those with less than a college education were much more likely to report receiving a care plan than those with more education. Survivors treated at a non-university based hospital were also more likely to receive a SCP than survivors treated at other healthcare settings. To better understand which of these demographic or cancer-related characteristics influenced whether a given patient received a SCP, we utilized random forest analysis to build a decision tree of the factors that influence whether a survivor received a SCP. The outcome variable for the random forest was whether a survivor received a SCP, a binary “yes” or “no.” Twenty four input variables were used for this analysis including continuous variables (diagnosis age, time since diagnosis, and number of treatments received), as well as categorical variables (including cancer type, race, and cancer treatment setting). A full list of input variables for the random forest model can be found in Supplemental Table 1.

**Figure 1:**
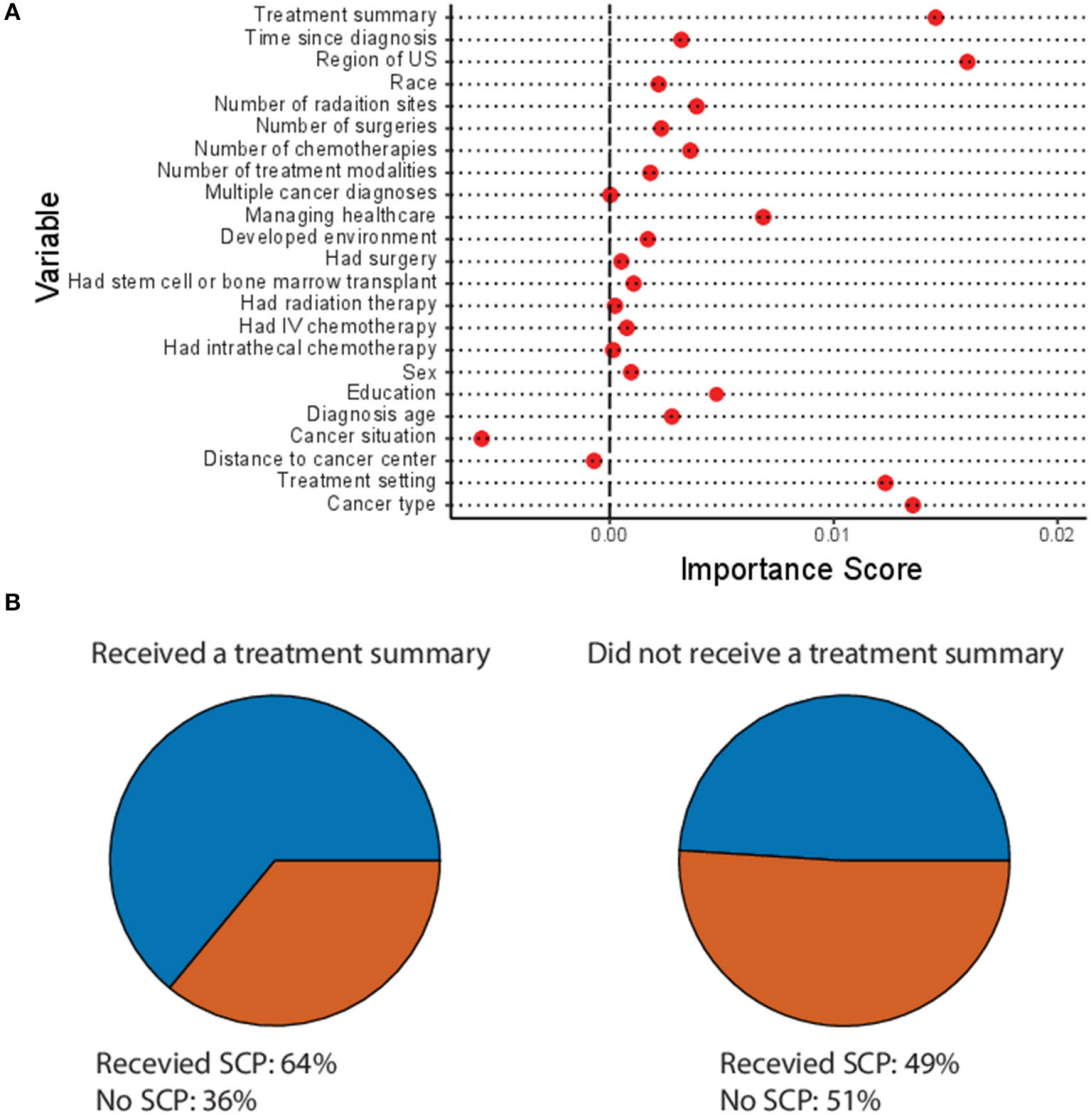
Predictors of survivorship care plan (SCP) use. **(A)** Variable importance plot of all variables from OncoLink included in the Random Forest analysis. Variables with a higher importance score have a larger impact in determining the accuracy of the model. **(B)** Proportion of survivors who received a treatment summary (left) or did not receive a treatment summary (right) who also did (blue) or did not (orange) receive a SCPs.

To guard against bias in the construction of the model, two-thirds of the total responses were used in building the random forest model and then the model was validated on the remaining one-third. The randomly selected sample input for each run of the random forest was weighted using the SEER cancer incidence rates (14), this was done to prevent the over-representation of breast cancer survivors in the dataset from biasing the results. The model was able to correctly identify whether or not a given survivor received a SCP 85% of the time. Additionally, each of the 24 variables was assigned an importance score, indicating the contribution of that variable to the overall accuracy of the predictive model (Figure 1A). Variables with a higher importance score are more influential in determining if a survivor will receive a SCP. The five most important variables in determining if a survivor will receive a care plan were cancer type, the setting in which they received their cancer care, the type of physician managing their care, whether they also received a treatment summary, and the geographic region in the United States in which they were treated (Figure 1A).

### Variation Between Cancer Types in Frequency of Survivorship Care Plan Use

Out of all cancer types in the study, breast cancer survivors received SCPs most frequently, with nearly 62% of breast cancer survivors receiving a plan (Supplemental Table 3). Rounding out the top three most frequent cancer types receiving care plans were lung and prostate cancer, both with 60% of survivors of each cancer type receiving plans. There was over a 20% difference in frequency of care plan use between the most and least served cancer types. The least frequently served survivor population, melanoma, received care plans <40% of the time. The next two lowest served cancer types were pancreatic cancer and liver cancer, both with <45% of survivors reporting receiving care plans (Supplemental Table 3).

While <15% of the respondents reported receiving a treatment summary, a separate document under the CoC guidelines containing just a list of treatments and dosages the survivor received, whether a survivor received a treatment summary also predicted whether they would receive a SCP (*p* < 0.00001 by *X*^2^ test). Survivors who received a treatment summary were significantly more likely to receive a SCP than those who did not (Figure 1B).

### Physician Type and Treatment Setting Influence Likelihood of Receiving SCPs

**Figure 2:**
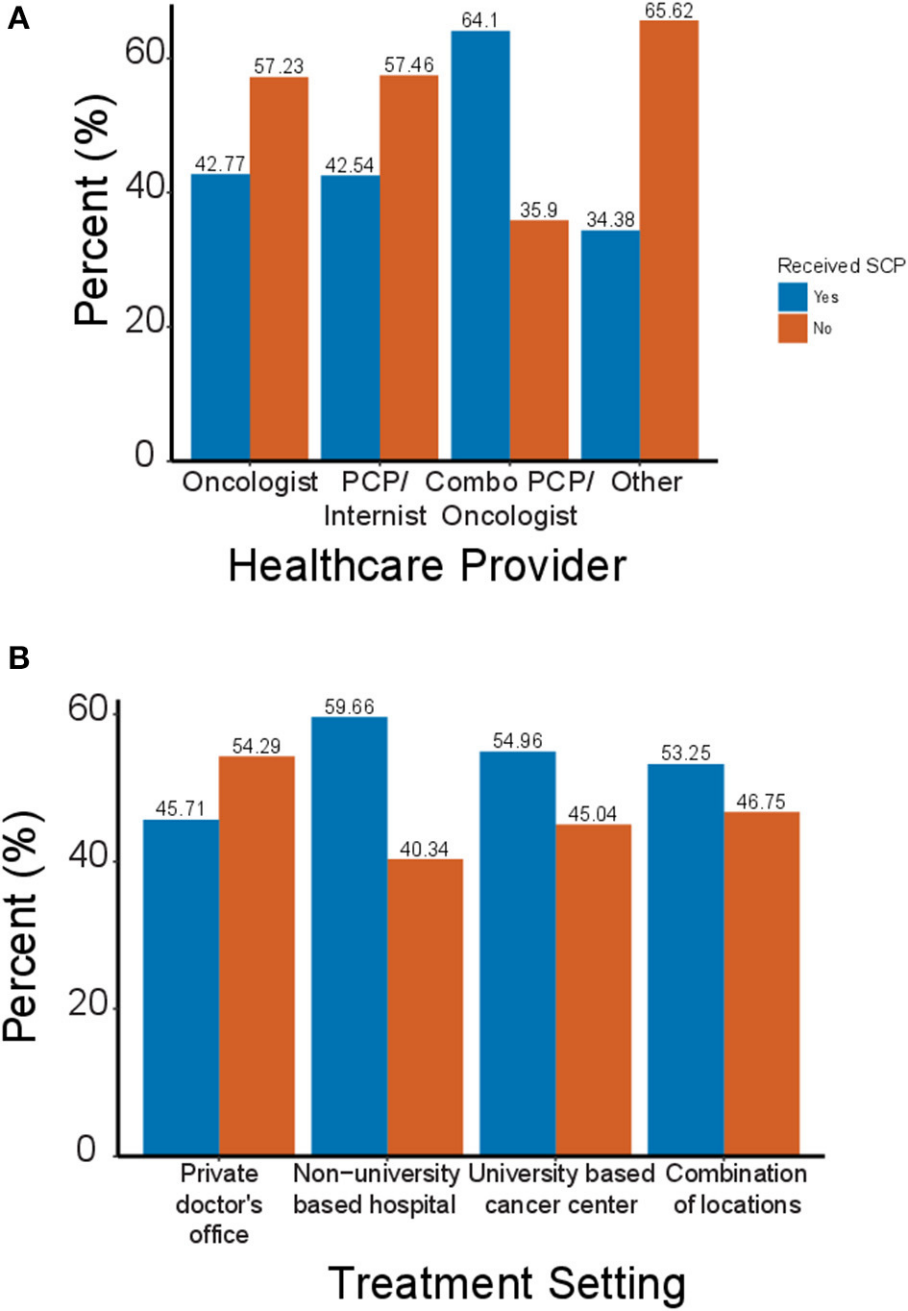
Survivorship care plan use by physician type and healthcare setting. **(A)** Percentage of cancer survivors who did (blue) or did not (orange) receive a SCP based on the type of physician who provided their cancer care. **(B)** Percent of cancer survivors who received a SCP based on the treatment setting in which they received care.

The type of physician who managed the survivors' care also strongly influenced their likelihood of receiving a SCP. Survivors who were treated by an oncologist or primary care physician were equally likely to receive a SCP, with 44 and 43% of survivors treated by each receiving care plans (Figure 2A). However, survivors treated by a combination of these two providers were significantly more likely to receive a care plan; 63% of survivors treated by a team of health care providers received a care plan. The lowest rates of SCP utilization were for survivors who were not treated by any of the above three options; these survivors received care plans <30% of the time.

In addition to the type of physician managing a survivor's care, the treatment setting in which a survivor received their cancer care also influenced the likelihood of receiving a SCP. Nearly 60% of survivors treated at university based cancer centers reported receiving a SCP (Figure 2B). Survivors treated at multiple sites or university based cancer centers received care plans at roughly equal rates of 53% each. The lowest rate of SCP utilization was for patients who were treated at a private doctor's offices; only 45% of survivors received a SCP.

### Regional Differences in Care Plan Utilization

**Figure 3:**
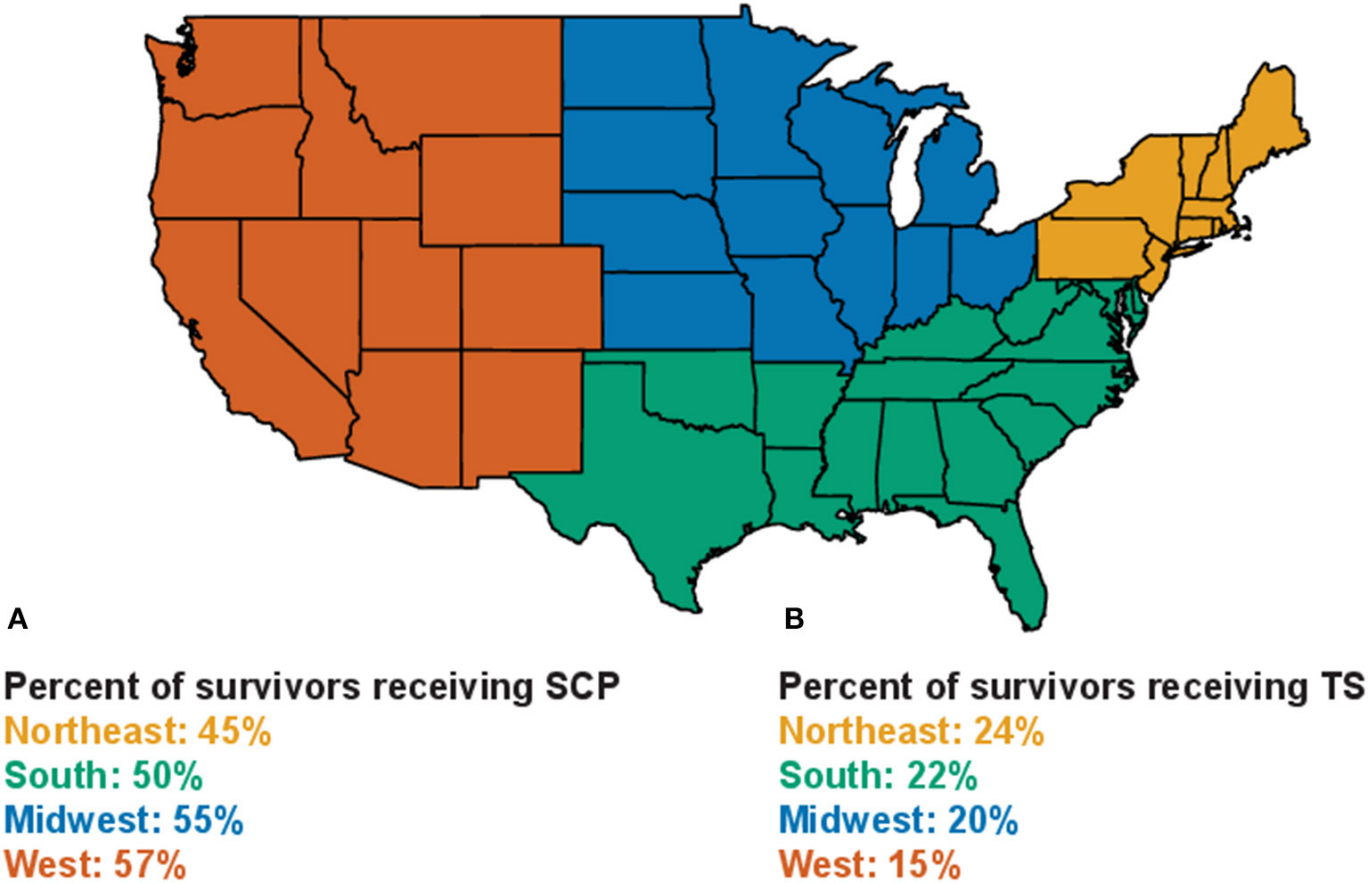
Patterns of treatment summary and SCP use by geographic region. **(A)** Percentage of survivors from each geographic region who reported receiving a SCP. **(B)** Percentage of survivors from each geographic region who reported receiving a treatment summary.

The geographic region in which the patients reported receiving treatment also significantly affected their likelihood of receiving a SCP (Table 1). While the number of responses from each region was similar, the frequency of SCP use varied significantly by region. Survivors in the northeast were the least likely to report receiving a SCP (Figure 3). Patients in the south were split 50/50 between those who received SCPs and those who did not. Survivors from the Midwest and West received SCPs at the highest rate, with 55 and 57% of survivors reporting receiving a plan, respectively. While many fewer survivors reported receiving a treatment summary as opposed to a SCP the patterns of use were opposite. Treatment summary use was highest in the Northeast at nearly 25% and lowest in the West with only 15% of survivors reporting receiving a treatment summary (Figure 3).

## Discussion

Survivorship care plans play an important role in bridging the transition from cancer patient to cancer survivor. SCPs improve patient knowledge, improve communication with their health care providers, and even decrease psychological problems (3–5). Despite strong calls for increased SCP use by groups such as the American Cancer Society, and efforts to tie SCP use to accreditation by groups such as the American College of Surgeons and the National Cancer Institute, SCP use in the U.S. remains low (7). This study utilized a national convenience sample representing the fifteen most common cancer types as well as many other less common malignancies to identify factors that influence whether a given cancer survivor will receive a SCP.

Despite having responses from all 50 states, this study was performed with a convenience sample of providers and survivors who utilized OncoLink, so the findings from this study should be validated in randomized patient populations or with other large databases of SCPs such as Journey Forward to ensure validity. Additionally, the assumption was made that all plans that were created ended up in the hands of the survivor they were created for. However, despite being a convenience sample, the sample population recaptured several survivorship trends previously observed in the literature including an over-representation of breast cancer survivors as well as low use of SCPs in private doctor's offices (7, 9). Additionally, survivors in this dataset were mostly female breast cancer patients and weighting the sample by cancer incidence type may not completely have balanced out this bias in our analysis.

Utilizing machine learning allowed us to build a decision tree to model how SCPs have been distributed across the United States over the last decade. The random forest analysis identified several factors that strongly influence whether a patient will receive/create a SCP, including patient characteristics (cancer type and where in the United States they live), as well as cancer care associated factors (type of physician managing their care, the healthcare setting where they received treatment, whether they received a treatment summary).

Consistent with literature showing that many hospitals in the United States use SCPs almost exclusively for breast and colorectal cancer patients (9), survivors of these two cancers were significantly more like to receive a care plan in our analysis as well. While these are among the most common cancer types and therefore an excellent place to start with SCPs, other common cancers such as melanoma and leukemia received care plans much less frequently. There were no other variables included in the data set that were able to explain this difference, although one missing feature is cancer subtype (i.e., BRAF-mutant melanoma) which has a much higher risk of recurrence.

The type of physician who managed survivors' cancer care strongly influenced whether a survivor would receive/create a SCP. Survivors who were managed by a combination of an oncologist and a primary care provider were over 20% more likely to receive a care plan than patients managed by just an oncologist or primary care provider alone. This is consistent with the pattern observed in a previous study of skin cancer patients (11). Since one of the goals of SCPs is to improve communication between the different health care providers managing a survivor's care, it is particularly encouraging that those survivors managed by a team received plans at such high rates. There are opportunities for improvement, however, since rates of care plan use by single providers were <50% for oncologists, PCPs, or other specialists.

Where patients received their care also significantly influenced their likelihood of receiving a SCP. While rates of SCP use were particularly low in private doctor's offices, no locations gave care plans to more than 60% of survivors. Care plan use also varied between different regions of the country. Despite OncoLink being housed at the University of Pennsylvania, respondents were equally split between the Northeast, South, Midwest, and West. This diverse representation could be because OncoLink has been featured in national magazines such as Readers Digest and by cancer support organizations such as the Livestrong Foundation and the American Cancer Society. Survivors from the Midwest and West were much more likely to receive/create care plans than those from the Northeast or South. Regional trends in care plan use were inversely related to treatment summary use, with the lowest frequency of treatment summaries being used in the West and the highest in the Northeast. This could be due to differences in priorities of survivorship programs, or as these documents can be given together could highlight an issue with question comprehension in the respondents.

This analysis highlights several inconsistencies in the way SCPs are used throughout the United States, from type of cancer patient, to where and by whom they were treated, there are disparities in who is or is not receiving a care plan. Identifying these underserved patient groups is an important step toward making sure they receive care plans. Targeting these underserved groups offers an avenue toward reaching the levels of SCP use necessary for accreditation by the American College of Surgeons. Another follow-up of this study would be to strive to collect information about what impacts the receipt/creation of a SCP had on the survivors.

## Data Availability Statement

The datasets generated for this study are available on request to the corresponding author.

## Ethics Statement

The studies involving human participants were reviewed and approved by University of Pennsylvania Perelman School of Medicine Institutional Review Board. The ethics committee waived the requirement of written informed consent for participation.

## Author Contributions

JB: conceptualization, methodology, software, formal analysis, investigation, writing–original draft, writing–review and editing, visualization, and project administration. CV, CB, KA-K, and MH: writing–review and editing and project administration. JM: resources, data curation, writing–review and editing, and project administration. CH-K: conceptualization, methodology, resources, data curation, writing–review and editing, supervision, and project administration.

### Conflict of Interest

The authors declare that the research was conducted in the absence of any commercial or financial relationships that could be construed as a potential conflict of interest.

## Supplementary Material

Supplemental Table 1 Variables from OncoLink included in Random Forest.

Supplemental Table 2 Breakdown of OncoLink users by cancer type.

Supplemental Table 3 Most and least frequent cancer types to receive survivorship care plans.
